# Impact of resolution, colour, and motion on object identification in digital twins from robot sensor data

**DOI:** 10.3389/frobt.2022.995342

**Published:** 2022-10-28

**Authors:** Paul Bremner, Manuel Giuliani

**Affiliations:** Bristol Robotics Laboratory, University of the West of England, Bristol, United Kingdom

**Keywords:** digital twins, robot, point clouds, voxels, user study

## Abstract

This paper makes a contribution to research on digital twins that are generated from robot sensor data. We present the results of an online user study in which 240 participants were tasked to identify real-world objects from robot point cloud data. In the study we manipulated the render style (point clouds vs voxels), render resolution (i.e., density of point clouds and granularity of voxel grids), colour (monochrome vs coloured points/voxels), and motion (no motion vs rotational motion) of the shown objects to measure the impact of these attributes on object recognition performance. A statistical analysis of the study results suggests that there is a three-way interaction between our independent variables. Further analysis suggests: *1*) objects are easier to recognise when rendered as point clouds than when rendered as voxels, particularly lower resolution voxels; *2*) the effect of colour and motion is affected by how objects are rendered, e.g., utility of colour decreases with resolution for point clouds; *3*) an increased resolution of point clouds only leads to an increased object recognition if points are coloured and static; *4*) high resolution voxels outperform medium and low resolution voxels in all conditions, but there is little difference between medium and low resolution voxels; *5*) motion is unable to improve the performance of voxels at low and medium resolutions, but is able to improve performance for medium and low resolution point clouds. Our results have implications for the design of robot sensor suites and data gathering and transmission protocols when creating digital twins from robot gathered point cloud data.

## 1 Introduction

In recent years the use of robots for inspection and maintenance has become increasingly prevalent. In many use cases data produced by such robots needs to be human understandable, i.e., when there is a requirement for there to be a human in the loop to make decisions on how to act upon this data, e.g., for action planning and/or robot teleoperation. Indeed, for our particular use case of nuclear decommissioning there is a safety requirement by the nuclear industry for there to be a human in the loop. One way in which this data can be made human understandable is through digital twins of the environment, rendered based on robot sensor data. We refer to such representations as digital twins as they are faithful digital reconstructions of real world objects, such that observers of the digital twin of the environment can make operational decisions there that can be relied upon for real world actions (e.g., robot teleoperation, radioactive object identification).

A human user of a VR based teleoperation system can then operate in a Virtual Environment (VE) composed of digital twins. The need for such twins is driven by robots operating in unknown environments in which a direct camera feed may be either unavailable or inadequate for proper scene understanding and effective teleoperation. It is therefore important that a user of this system is able to recognize features of this digital twin environment such that they can operate effectively. How the environment is rendered will have a direct impact on this.

An important factor to consider in the rendering of digital twins is the availability of data. The sensors typically used to capture environmental features that can be used for digital twins, laser scanners and RGB-D cameras, generate point clouds from which digital twins can be composed. The significant size of point cloud data makes the transmission difficult and slow (Zhou et al., 2020). Further, there are many factors that affect the density of the resultant point clouds, for example, scanner specifications, data gathering time, communication bandwidth, and environmental features (smoke, radiation *etc*.). Additionally, there may be issues of computational overhead (which can cause frame rate reduction) in rendering large point clouds, such as might be required for a digital twin of a large environment. Consequently, it is important to understand the implications of reduced density point clouds on the human ability to recognise digital twins of environmental objects.

**FIGURE 1:**
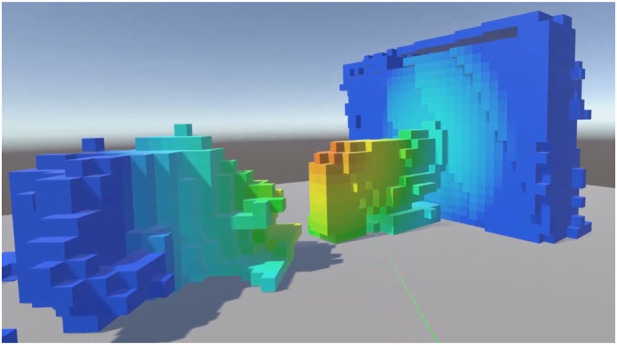
A voxel environment coloured by the levels of radiation at each point using a heat map paradigm.

The capability to render digital twins in colour, another likely factor in object recognition, is also reliant on data availability. In recent systems where colour information is available it is utilised to add fidelity to the rendered virtual environment (Mossel and Kroeter, 2017; Giorgini and Aleotti, 2018; Stotko et al., 2019; Valenzuela-Urrutia et al., 2019). However, in addition to the factors affecting the density with which point clouds can be rendered listed above, point clouds may need to be coloured to display sensor information present at each point (rather than visible colour) such as temperature or radiation. A common use of artificial point colouring is to display point elevation (Bergé et al., 2016; Schwarz et al., 2017). Figure 1 shows a point cloud coloured in this way. Consequently it is important to understand how colour impacts human identification of digital twins of environmental objects.

Quantifying the impact of colour and point cloud density will inform the selection of sensors, data gathering procedures, data communication protocols, and facilitate the minimisation of computational overhead for rendering (to maximise frame rates when navigating in the virtual environment).

Faithful rendering of point cloud data results in the highest fidelity digital twins. Indeed, it is a typical and reasonable approach for remote scene reconstruction for dynamic scenes where real-time point cloud to model rendering is almost impossible (Lesniak and Tucker, 2018). However, doing so is not without issue. The apparent solidity of point cloud objects is dependent on viewer distance, as an object is neared it can appear to dissipate for the viewer (Bruder et al., 2014). Perhaps more importantly for robot teleoperation and operational planning, point cloud objects cannot be natively selected and manipulated using standard ray-based methods (Bruder et al., 2014). Relatedly, in many virtual environment rendering engines occlusion culling for point clouds is a challenging and memory intensive problem often resulting in more points than necessary being rendered (even when invisible to an observer) unnecessarily increasing computational overhead. It has been found that as computational overhead goes up the data refresh rate must be slowed to ensure a smooth control experience (Codd-Downey et al., 2014).

A common solution to the aforementioned issues is to use a voxel representation of point cloud data (Mossel and Kroeter, 2017; Zhou and Tuzel, 2018; Li et al., 2022). A voxel is a 3D pixel, i.e., a cube of fixed dimensions arranged as part of a complete 3D grid. A voxel is visible if it contains within its bounds sufficient point cloud points (typically set at some threshold to reduce noise effects).

Voxel colour is some function of the colours of points it represents. As with point clouds voxels might need to be coloured in order to display data. Importantly as 3D virtual objects voxels are subject to scene lighting effects in the virtual environment and this impacts how they are perceived by users in the virtual scene.

Voxels effectively down-sample point cloud data, while maintaining some degree of fidelity. This down-sampling may impact the ability of users to recognise objects represented in this way. Coupled with the utility that voxels provide, it is important to quantify the impact voxelisation might have on point cloud data.

Here we have only considered voxels as a rendering alternative to point clouds, opting not to include automatic mesh generation as a rendering alternative. We have made this decision as voxels are relatively unaffected by lower point cloud density, maintain the veracity of the data (which can be distorted with mesh generation), and are less computationally intensive. These factors are of high importance in our use case where high data refresh rates are necessary for safe robot teleoperation.

As the purpose of environmental digital twins is rendering a virtual environment, users of such a system are able to move relative objects in the environment. Motion is a fundamental part of the human visual system, aiding our ability to perceive our environment (Nakayama, 1985). It therefore seems reasonable to expect that motion will aid in the identification of digital twins, and that it might compensate for limitations in available data.

In this paper we report the results of a user study that has allowed us to evaluate and quantify the impact of rendering approach (point clouds of different densities, and voxels of different granularities), colour (monochrome vs coloured), and motion (no motion vs rotational motion) in digital twins that are generated from robot point cloud data. The study aims to evaluate the effects of representation type, resolution, colour and motion on the identification of digital twins of real world objects, and the interplay between these factors. In the context of point clouds we define resolution as point cloud density, and for voxels we define it to mean the granularity of the voxel grid (determined by voxel edge length)^1^. As detailed above these factors are key in determining rendering and interface design choices for digital twin virtual environments, thus understanding of their impact will aid future system design.

## 2 Literature review

Rendering digital twins of the environment from robot sensor data is a relatively new field, hence there is limited literature in it. An early proponent of creating Point Cloud Virtual Environments (PCVEs) was Bruder et al. (2014). They proposed a novel approach for rendering a PCVE from robot sensor data. A number of authors have extended this idea using different approaches to render PVCEs (Schwarz et al., 2017; Lesniak and Tucker, 2018; Valenzuela-Urrutia et al., 2019), Voxel Virtual Environments (VVEs) (Mossel and Kroeter, 2017; Zhou and Tuzel, 2018; Stotko et al., 2019; Li et al., 2022), and VEs composed of precomposed objects based on automated recognition of objects from the point cloud data (Zhou et al., 2020).

Common features of all of this prior work are: assumption on the availability of high density point cloud data, accurate colour information is also often assumed; description of a technical implementation, but with little or no user study to evaluate the efficacy of the system in use. These common features highlight the need for a large scale user study to evaluate the utility of rendering VEs as point clouds or voxels, and, in light of issues addressed in the introduction, the need to understand the impact of reductions in rendered object resolution.

An area of research which has examined object recognition of objects from sparse point clouds (described as random dots therein) is psychophysics (Sperling et al., 1989; Van Damme et al., 1994). In the psychophysics literature random dots are composed to display simple geometric shapes, and used to investigate the kinetic depth effect (how motion cues aid vision). However, as noted in Van Damme et al. (1994) the features of the shape being rendered effects the ease with which it can be recognised. This highlights the need to evaluate object recognition using objects with a high degree of ecological validity. Further, psychophisics studies perform in-depth testing of a small number of participants, in order to generate data that can be tested with robust statistics and be generalisable to a broad population, we have carried out a large scale user study.

More recent research has utilised the motion component of human vision in VE. Lubos et al. (2014) and Garrido et al. (2021) present a method by which point cloud data might be manipulated by a user, i.e., moving the point cloud around to allow better feature identification. Again these papers present a technical implementation without an evaluatory user study. Their work highlights the potential utility of motion in digital twin object identification, further motivating us to include motion as one of the features to be investigated.

## 3 Hypotheses

The user study presented here aims to test the following hypotheses:

H1. As the resolution of point clouds increases object recognition will improve.
H2. Point clouds will be easier to recognise than voxels.
H3. As the resolution of voxels increases object recognition will improve.
H4. Colour will have a greater impact for point clouds than for voxels.
H5. Motion will have a greater impact for point clouds than for voxels.
H6. Motion will compensate for a lack of colour.
H7. Motion will compensate for reduced resolution in point clouds but not voxels.

The basis for H1 is that as a higher fidelity representation of the data, point clouds will be easier to recognise than voxels. The visibility of details relating to the precise shape and colouring of objects is reduced as a consequence of the voxelisation process. Indeed, it is this reduction in fidelity of reproduction that is the basis for H2 and H3. In the commonly used KITTI benchmark data set the object recognition difficulty is determined by the resolution of the point cloud: lower resolution means harder recognition (Geiger et al., 2013). While this determination is based on artificial classifiers, it seems likely that it will hold to some degree for human recognition from point clouds. While not included in this data set it seems reasonable to conclude that the same is likely to be true for voxel object recognition.

Colour information is utilised to delineate between different elements in an image in a variety of applications (Patel et al., 2012). However, in the presence of shading information human recognition of images is unaffected by a lack of colour (Kemp, 1996). These findings underpin H4: voxels are subject to lighting effects, hence colour is less necessary to delineate between different parts of an object, and thus will have a bigger impact on point clouds than voxels.

As described in the introduction, movement forms a fundamental part of human visual processing. Of particular relevance here is the kinetic depth effect, which enables an observer to recover an objects 3D structure as result of the fact that different parts of an object appear to move in different directions relative to the observer, as it moves relative to them (Hale and Stanney, 2014). This effect allows people to more easily recognise shapes in monochrome dot clouds (Sperling et al., 1989). Studies with random 3D dot patterns found that motion enabled observers to distinguish between dots which they were otherwise unable to do (Nakayama, 1985). Similarly Hardy et al. (1996) found that rotation was important for recognition of 3D X-ray images. These findings underpin H5-7: voxels are subject to lighting effects hence motion is less necessary to delineate between different parts of an object, and thus will have a bigger impact on point clouds than voxels. Further, for H6, the shading in voxels will compensate to some degree if there is an absence of colour information, aiding delineation of different component parts of the objects, hence motion will provide a stronger compensatory effect for point clouds than for voxels. H7 follows a slightly different line of reasoning in that motion will provide more detail and contextual cues for the make up of the digital twin objects for point clouds, but the downsampling process of reduced resolution voxels means limited additional information will be provided by rotation.

## 4 Study design

In order to evaluate our hypotheses we needed to ensure participants observed data consistently, as well as having a large sample size, thus we developed an online user study with pre-rendered images of our digital twins. Our user study follows a mixed design with render (three resolutions of point clouds and three resolutions of voxels) as the between subjects condition and colour (colour or monochrome) and motion (static or rotating) as the within subjects conditions. The study was conducted on the Qualtrics survey platform^2^ and 240 participants (125 male, 104 female, 11 unknown, age M 26.9 SD 8.1) were recruited *via* the Prolific Academic recruitment portal^3^. Average study duration was 19 min, and participants were compensated *£*2.45 for their time. The study was approved by the University of the West of England ethics committee.

### 4.1 Stimulus design

**FIGURE 2:**
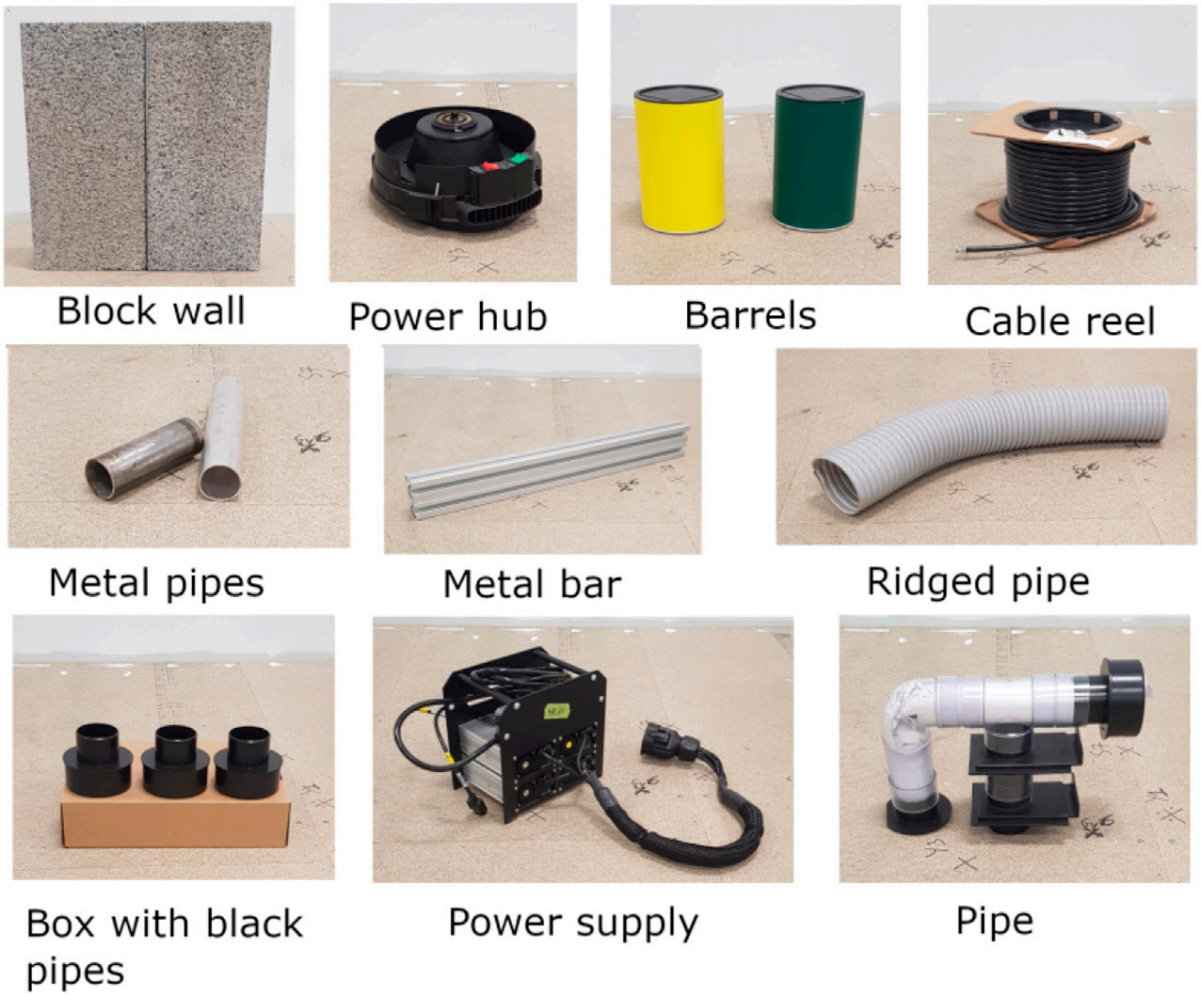
The named object picture displayed for each trial in the questionnaire.

To construct the stimulus material scale versions of industrial objects (such as might be found on a nuclear decommisioning site) were assembled (*see* Figure 2). These objects were captured using a RealSense D415 RGB-D camera. Output from the camera was input to the RtabMap ROS package^4^ that automatically registers multiple point clouds into a single point cloud map. By panning the camera around each object, a complete point cloud representation of each object was created and exported to a file; each point in the file had a RGB values in addition to XYZ co-ordinates.

**FIGURE 3:**
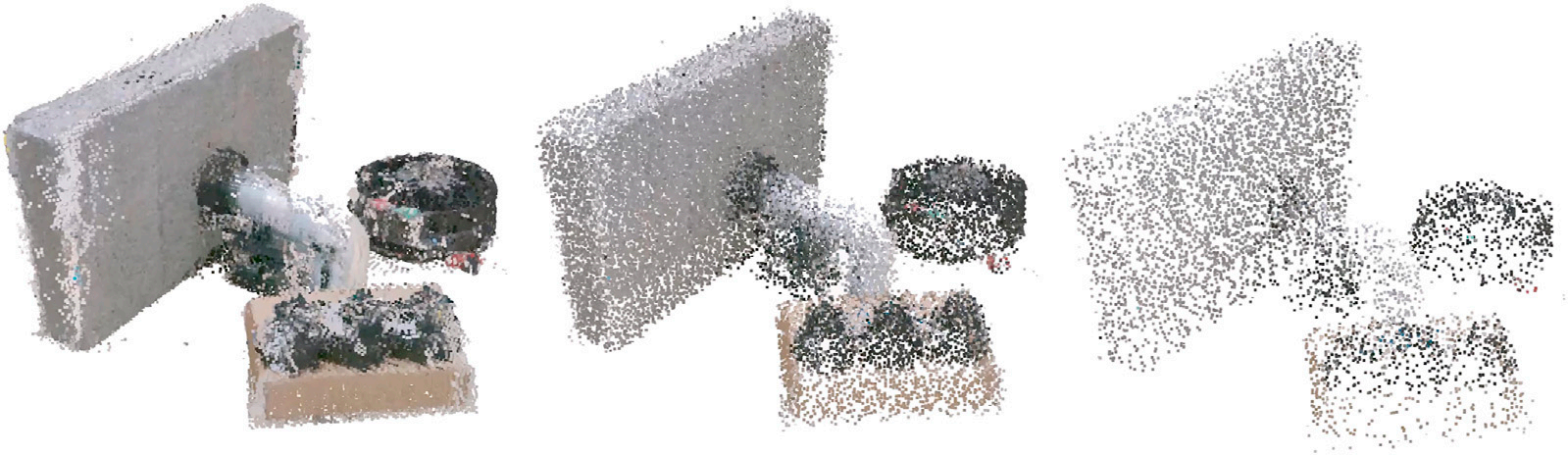
An example scene rendered at the three resolutions of point clouds.

The point cloud files were individually imported into Unity for rendering. In order to import the point clouds a set of Unity scripts were written based upon the free-point-cloud-viewer Unity asset^5^, so that points were arranged into logical mesh chunks according to their location, and only points within a pre-defined bounding box were rendered to remove noise (i.e., points outside the object). An initial render of each point cloud was used to define the bounding box for each object. Three resolutions (densities) of point cloud were individually rendered and saved as assets for later scene composition. To create the different densities the rendering script was modified to display all points (PC1), every eighth point (PC8), and every 35th point (PC35); the densities were selected empirically to give three distinct resolutions, high, medium and low. Figure 3 shows example scenes in the three resolutions.

**FIGURE 4:**
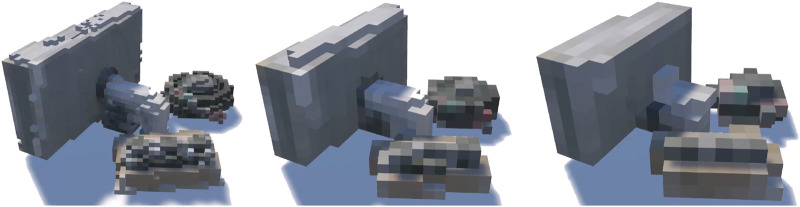
An example scene rendered at the three resolutions of voxels.

To create the voxel versions of the captured objects a rendering script was written that divided the bounding box for each object into a 3D grid, and rendered a voxel at each grid position containing a sufficient number of points (empirically set to five points to exclude noise, but render all component voxels). Each voxel was coloured according to the mean of the RGB values of the points contained within that voxel. Three resolutions (granularities) of voxel were individually rendered and saved as assets for later scene composition. To create the three voxel resolutions three different voxel edge lengths were specified 0.2 (VX2), 0.4 (VX4), and 0.6 (VX6) Unity world units. As with the point clouds these voxel granularities were selected empirically to give three distinct resolutions, high, medium and low. Figure 4 shows example scenes in the three resolutions.

**FIGURE 5:**
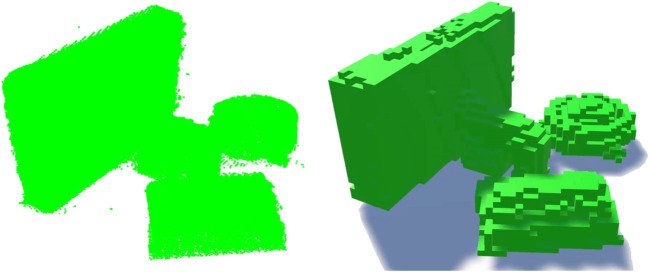
An example scene rendered in monochrome as a high resolution point cloud and high resolution voxels.

Twelve scenes were created with three or four objects pseudo-randomly selected, such that no scene contained more than two of the same object, and how many objects to expect was unknown to participants. Objects were placed in close proximity on a white plane such that there was some overlap between objects when viewed from an isometric viewpoint selected for the static images. Figure 3 shows an example scene. Each scene was created in all six rendering conditions, and had a single light source such that shadows were created in the voxel scenes. Monochrome versions of each scene were created by setting the colour of all points to green: this ensured they were distinct from the base plane regardless of lighting effects or display settings (of participant’s monitors). Figure 5 shows example monochrome scenes.

The motion condition was created by recording the scenes rotating around a central point (converted to GIFs for embedding in the questionnaire, example GIF included in the Supplementary Material). This rotation is somewhat analogous to users of a virtual environment panning around an object for examination while keeping it in view (as we observed participants doing in ?). Moreover, it matches the motion utilised in the psychophysics literature investigating the kinetic depth effect (Nakayama, 1985; Sperling et al., 1989; Hale and Stanney, 2014).

### 4.2 Questionnaire design

Using the Qualtrics survey builder an identical version of the questionnaire was built for each of the rendering conditions. Participants first saw an explanation of the identification task: select how many objects are present in each image, and then specify, for each object, where in the image it appears, and select the object from the set of possible objects shown in the image (Figure 2, not all objects shown appeared in the scenes, this image was available on each experimental trial). Participants answered three trials per motion/colour combination, for a total of twelve trials, presented in a random order. Each of the twelve stimulus scenes was randomly assigned to a different trial, so no scene was repeated, and each participant saw a different combination of conditions and scenes.

For the motion conditions participants were shown both the rotating image and the static image so that they could specify locations for each object they identified. This is somewhat analogous to being in the virtual environment where motion can be controlled, i.e., the results will still be applicable to the use of digital twins in actual virtual environments.

In the Prolific Academic recruitment portal participants were constrained to only be able to participate in one study condition.

## 5 Results

The results are processed to calculate the mean percentage correct object identifications for each participant over the three trials that they performed for each condition. The results ignore the object location participants were asked to complete: ambiguities in the position labels for some of the scenes meant that the data was unreliable, often objects would be correctly identified but the position would be slightly wrong (e.g., back left for an object that was centre left as there were no objects in the back row).

The data was analysed using R to perform a three-way mixed ANOVA, and follow up statistical tests^6^. The results of the three-way Anova are shown in Table 1, significant main effects were found for all three conditions, as well as all two-way and the three-way interactions being significant. The key result here is that there is a three way interaction between the conditions, in the following sections we decompose this interaction for analysis.

**TABLE 1:** 3-Way Mixed ANOVA results showing main effects and interactions.

Effect	DFn	DFd	F	*p*
Render	5	226	55.538	1.78e−37*
Colour	1	226	55.962	1.63e−12*
Motion	1	226	121.345	7.20e−23*
render:colour	5	226	3.376	6.00e−03*
render:motion	5	226	11.307	9.57e−10*
colour:motion	1	226	69.275	8.16e−15*
render:colour:motion	5	226	18.628	1.73e−15*

Significant results where *p* < 0.05 indicated with *.

**FIGURE 6:**
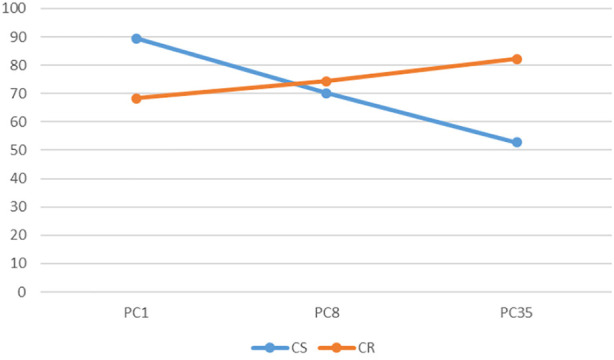
The render:motion interaction for point clouds in colour.

**FIGURE 7:**
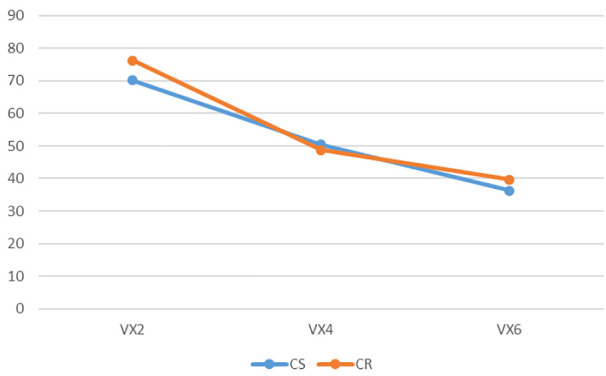
The render:motion interaction for voxels in colour.

**FIGURE 8:**
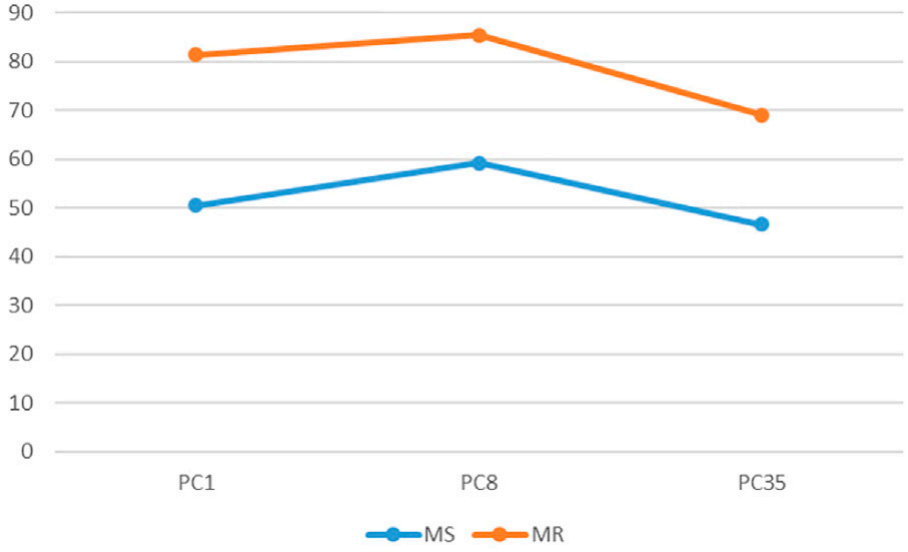
The render:motion interaction for point clouds in monochrome.

**FIGURE 9:**
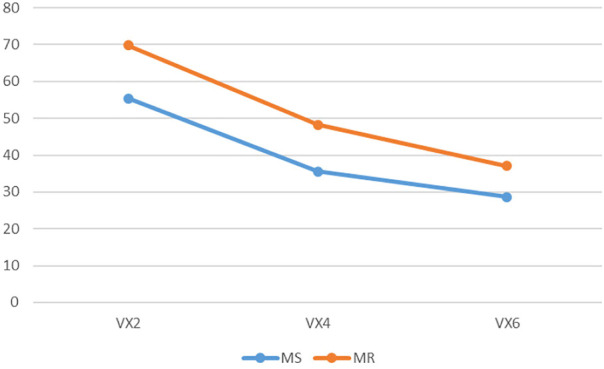
The render:motion interaction for voxels in monochrome.

**FIGURE 10:**
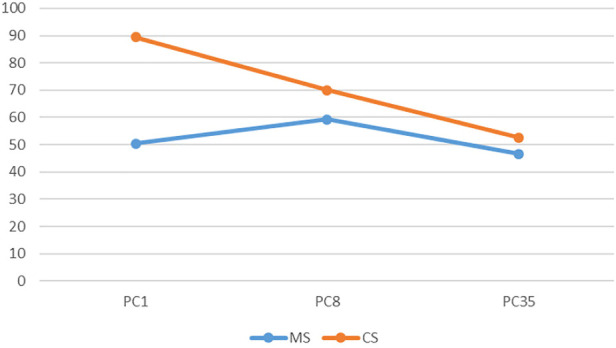
The colour:motion interaction for static point clouds.

**FIGURE 11:**
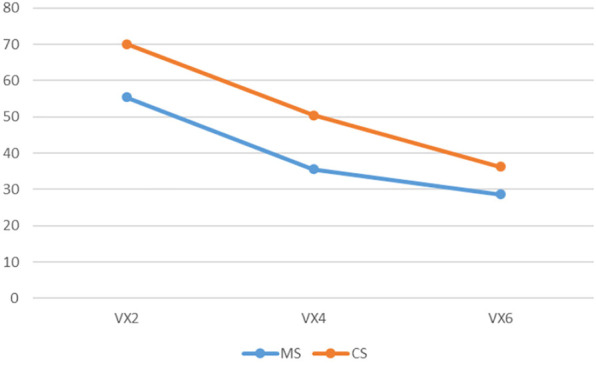
The render:motion interaction for static voxels.

**FIGURE 12:**
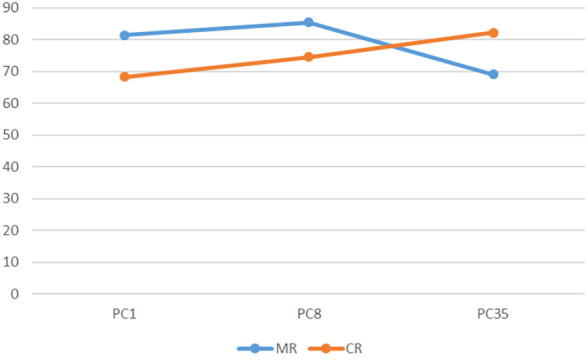
The render:motion interaction for rotating point clouds.

**FIGURE 13:**
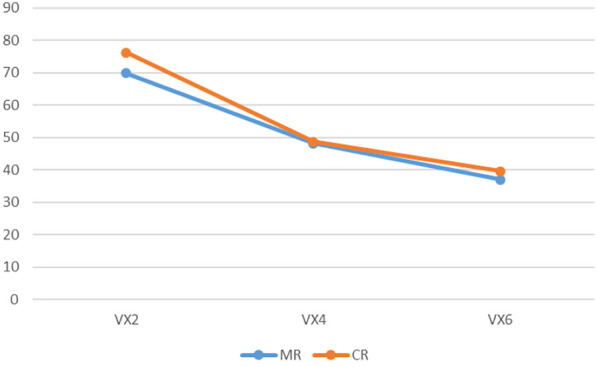
The render:motion interaction for rotating voxels.

To analyse the significant three-way interaction simple two-way interactions were calculated for render:motion and render:colour. The results are shown in Tables 2, 3. In both cases the key result is that the two-way interactions were found to be significant across both levels of the third variable at Bonferroni adjusted *p* < 0.025. To better illustrate the two-way interactions we have produced a set of interaction graphs, Figures 6–13.

**TABLE 2:** Simple two way interactions of render:motion across both levels of colour, Colour and Monochrome.

Colour	Effect	DFn	DFd	F	*p*
C	render	5	229	46.7	3.83e−33*
C	motion	1	229	6.11	1.4e−2*
C	render:motion	5	229	24.3	1.27e−19*
M	render	5	232	34.6	2.17e−26*
M	motion	1	232	186	1.61e−31*
M	render:motion	5	232	6.85	5.58e−6*

Significant results where *p* < 0.025 (Bonferroni adjusted) indicated with *.

**TABLE 3:** Simple two way interactions of render:colour across both levels of motion, Rotating and Static.

Motion	Effect	DFn	DFd	F	*p*
R	render	5	227	61.9	1.65e−40*
R	colour	1	227	0.22	6.40E−01
R	render:colour	5	227	10.2	7.21e−9*
S	render	5	234	29.8	2.3e−23*
S	colour	1	234	124	2.43e−23*
S	render:colour	5	234	12.2	1.72e−10*

Significant results where *p* < 0.025 (Bonferroni adjusted) indicated with *.

In the colour condition as point cloud resolution decreases recognition decreases for static objects but increases for objects in motion (Figure 6). For voxels the impact of motion varies with resolution: at medium and low resolution motion has less effect than at high resolution (Figure 7). In the monochrome condition for point clouds motion has a larger effect at high resolution than at medium and low resolutions. Also of note is that the change of resolution from high to medium results in a larger effect in the static condition than in the rotation condition (Figure 8). For voxels performance change is similar across resolutions, though at low resolution there is less improvement with motion (Figure 9).

In the static condition for point clouds colour has a much higher impact at high resolution, compared to a minimal impact at medium and low resolutions. Also of note is that as resolution decreases there is a decrease in performance for colour point clouds, where as performance at low and high resolutions is similar for monochrome point clouds, with an increase in performance at medium resolution (Figure 10). For voxels, colour has a similar effect at high and medium resolutions, but decreases in efficacy at low resolution (Figure 11). In the rotation condition for point clouds colour has similar impact at high and medium resolutions, but at low resolution performance sharply decreases for monochrome point clouds but increases for colour point clouds (Figure 12). For voxels, colour only produces a small increase in performance at high resolution and has almost no impact at low and medium resolutions (Figure 13).

To decompose the significant interactions render:motion and render:colour we have calculated simple simple main effects for render across all colour and motion conditions. The simple simple main effect of render was significant for all combinations of colour and motion, thus indicating how the data is rendered is significant in all these cases. The results are shown in Table 4.

**TABLE 4:** Simple simple main effect of render.

Colour	Motion	Effect	DFn	DFd	F	*p*
C	R	render	5	231	39.0	5.91e−29*
M	R	render	5	232	43.7	1.26e−31*
C	S	render	5	234	39.2	3.69e−29*
M	S	render	5	236	12.2	1.62e−10*

Significant results where *p* < 0.0125 (Bonferroni adjusted) indicated with *.

**FIGURE 14:**
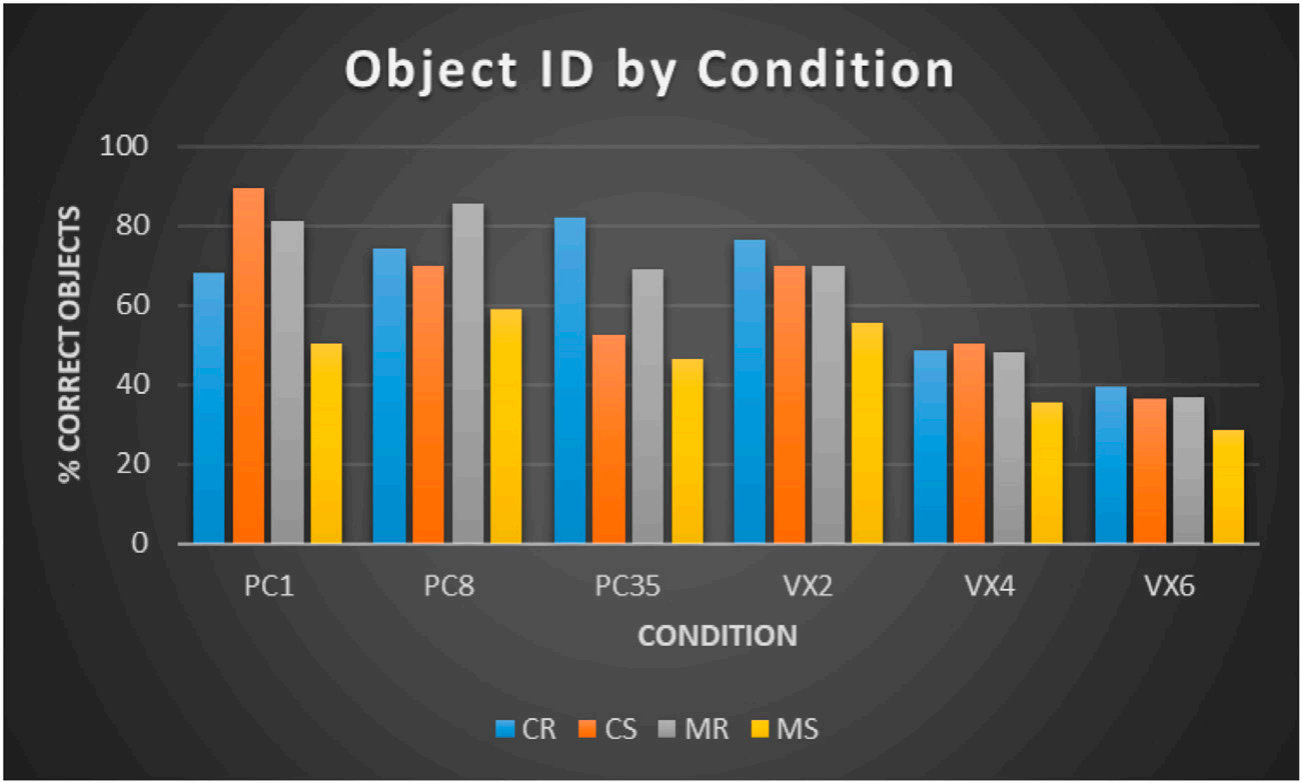
The mean percentage of correct object identifications across all conditions. PC, Point Cloud; VX, Voxel. Resolution for each render style decreases from left to right. C, Colour; M, Monochrome; R, Rotation; S, Static.

To decompose the simple simple main effects of render we have calculated simple simple pairwise comparisons were run for each motion and colour condition across all levels of the other conditions, *p* values were adjusted using Bonferroni correction. The results are shown in Table 6 and Table 7. Summaries of key statistics pertaining to the proposed hypotheses follow. The data underlying these pairwise comparisons is shown in Figure 14.

**TABLE 5:** Pairwise render comparisons.

Colour	Motion	group1	group2	n1	n2	Statistic	df	*p*	p.adj
C	R	PC1	PC35	40	40	−3.5549569	39	1.00E−03	1.50E−02*
C	R	PC1	PC8	40	40	−1.3659849	39	1.80E−01	1.00E+00
C	R	PC1	VX2	40	40	−1.9969039	39	5.30E−02	7.92E−01
C	R	PC1	VX4	40	40	4.5328175	39	5.40E−05	8.10E−04***
C	R	PC1	VX6	40	40	6.5290408	39	9.56E−08	1.43E−06****
C	R	PC8	VX2	40	40	−0.8005131	39	4.28E−01	1.00E+00
C	R	PC8	VX4	40	40	4.9728769	39	1.36E−05	2.04E−04***
C	R	PC8	VX6	40	40	8.9517366	39	5.34E−11	8.01E−10****
C	R	PC35	PC8	40	40	2.1287393	39	4.00E−02	5.94E−01
C	R	PC35	VX2	40	40	1.2357095	39	2.24E−01	1.00E+00
C	R	PC35	VX4	40	40	7.0746476	39	1.69E−08	2.54E−07****
C	R	PC35	VX6	40	40	10.3327348	39	1.00E−12	1.50E−11****
C	R	VX2	VX4	40	40	6.4611644	39	1.19E−07	1.78E−06****
C	R	VX2	VX6	40	40	9.2473611	39	2.24E−11	3.36E−10****
C	R	VX4	VX6	40	40	1.9257072	39	6.20E−02	9.22E−01
C	S	PC1	PC35	40	40	9.3045261	39	1.89E−11	2.84E−10****
C	S	PC1	PC8	40	40	5.4389377	39	3.11E−06	4.66E−05****
C	S	PC1	VX2	40	40	3.8877231	39	3.83E−04	6.00E−03**
C	S	PC1	VX4	40	40	10.3253183	39	1.03E−12	1.55E−11****
C	S	PC1	VX6	40	40	13.8416573	39	1.24E−16	1.86E−15****
C	S	PC8	VX2	40	40	−0.3613254	39	7.20E−01	1.00E+00
C	S	PC8	VX4	40	40	4.7041023	39	3.17E−05	4.75E−04***
C	S	PC8	VX6	40	40	6.8102794	39	3.91E−08	5.86E−07****
C	S	PC35	PC8	40	40	−3.7726311	39	5.37E−04	8.00E−03**
C	S	PC35	VX2	40	40	−3.5757021	39	9.51E−04	1.40E−02*
C	S	PC35	VX4	40	40	0.4804497	39	6.34E−01	1.00E+00
C	S	PC35	VX6	40	40	3.494462	39	1.00E−03	1.80E−02*
C	S	VX2	VX4	40	40	4.8812217	39	1.82E−05	2.73E−04***
C	S	VX2	VX6	40	40	8.4130699	39	2.68E−10	4.02E−09****
C	S	VX4	VX6	40	40	3.5152592	39	1.00E−03	1.70E−02*
M	R	PC1	PC35	40	40	3.2627296	39	2.00E−03	3.40E−02*
M	R	PC1	PC8	40	40	−0.9245654	39	3.61E−01	1.00E+00
M	R	PC1	VX2	40	40	2.1212116	39	4.00E−02	6.05E−01
M	R	PC1	VX4	40	40	7.3929615	39	6.22E−09	9.33E−08****
M	R	PC1	VX6	40	40	11.1837269	39	9.81E−14	1.47E−12****
M	R	PC8	VX2	40	40	3.4209934	39	1.00E−03	2.20E−02*
M	R	PC8	VX4	40	40	8.1384375	39	6.19E−10	9.28E−09****
M	R	PC8	VX6	40	40	12.7363707	39	1.79E−15	2.68E−14****
M	R	PC35	PC8	40	40	−4.1263255	39	1.87E−04	3.00E−03**
M	R	PC35	VX2	40	40	−0.3830965	39	7.04E−01	1.00E+00
M	R	PC35	VX4	40	40	3.8293739	39	4.54E−04	7.00E−03**
M	R	PC35	VX6	40	40	6.7498512	39	4.73E−08	7.10E−07****
M	R	VX2	VX4	40	40	4.7330588	39	2.90E−05	4.35E−04***
M	R	VX2	VX6	40	40	9.03987	39	4.12E−11	6.18E−10****
M	R	VX4	VX6	40	40	2.8293887	39	7.00E−03	1.10E−01
M	S	PC1	PC35	40	40	0.9557043	39	3.45E−01	1.00E+00
M	S	PC1	PC8	40	40	−1.9236397	39	6.20E−02	9.25E−01
M	S	PC1	VX2	40	40	−1.1484918	39	2.58E−01	1.00E+00
M	S	PC1	VX4	40	40	3.4164084	39	2.00E−03	2.20E−02*
M	S	PC1	VX6	40	40	5.1822505	39	7.04E−06	1.06E−04***
M	S	PC8	VX2	40	40	0.4565612	39	6.51E−01	1.00E+00
M	S	PC8	VX4	40	40	4.713054	39	3.08E−05	4.62E−04***
M	S	PC8	VX6	40	40	7.1811758	39	1.21E−08	1.82E−07****
M	S	PC35	PC8	40	40	−2.9568625	39	5.00E−03	7.90E−02
M	S	PC35	VX2	40	40	−2.1374752	39	3.90E−02	5.83E−01
M	S	PC35	VX4	40	40	2.0396524	39	4.80E−02	7.23E−01
M	S	PC35	VX6	40	40	4.2971382	39	1.12E−04	2.00E−03**
M	S	VX2	VX4	40	40	3.718221	39	6.29E−04	9.00E−03**
M	S	VX2	VX6	40	40	6.5161235	39	9.96E−08	1.49E−06****
M	S	VX4	VX6	40	40	1.5783085	39	1.23E−01	1.00E+00

Significance indicated by *s for Bonferroni adjusted *p* values.

In order to address H1, H4 and H5 we compare point clouds and voxels in each colour-motion condition separately. For colour-rotation, point clouds at all three resolutions perform equally as well as the high resolution voxels, but significantly better than voxels at medium and low resolutions. For colour-static: point clouds at high resolution are significantly better than voxels at all resolutions; point clouds at medium resolution are significantly better than medium and low resolution voxels; low resolution point clouds are significantly better than low resolution voxels, but significantly worse than high resolution voxels. For monochrome-motion, point clouds at all three resolutions significantly outperform medium and low resolution voxels, but only medium resolution point clouds significantly outperform high resolution voxels. For monochrome-static, medium and high resolution point clouds significantly outperform medium and low resolution voxels, the low resolution point clouds only significantly outperform the low resolution voxels.

**TABLE 6:** Pairwise motion comparisons.

Colour	Render	group1	group2	n1	n2	*p*	p.adj
C	PC1	R	S	40	40	2.47E−07	2.47E−07****
M	PC1	R	S	40	40	9.57E−11	9.57E−11****
C	PC8	R	S	40	40	3.04E−01	3.04E−01
M	PC8	R	S	40	40	1.04E−08	1.04E−08****
C	PC35	R	S	40	40	4.62E−11	4.62E−11****
M	PC35	R	S	40	40	1.80E−07	1.80E−07****
C	VX2	R	S	42	42	8.80E−02	8.80E−02
M	VX2	R	S	42	42	7.89E−05	7.89E−05****
C	VX4	R	S	40	40	6.32E−01	6.32E−01
M	VX4	R	S	40	40	4.00E−03	4.00E−03**
C	VX6	R	S	40	40	3.08E−01	3.08E−01
M	VX6	R	S	40	40	1.20E−02	1.20E−02*

Significance indicated by *s for Bonferroni adjusted *p* values.

In order to address H4 and H5 we compare across resolutions within each render style (point clouds and voxels) in each colour-motion condition separately. Point cloud performance significantly decreases with resolution in the colour-static condition, but is only significantly different between high and low resolutions in the colour-rotation condition, where it increases. For monochrome-rotation high and medium resolution point clouds perform significantly better than low resolution point clouds, where as for monochrome-static performance is not significantly different across all three resolutions. High resolution perform significantly better than medium and low resolution voxels in all colour-motion combinations. Medium resolution voxels perform significantly better than low resolution voxels in the colour-static condition but are not significantly different in the other conditions.

**TABLE 7:** Pairwise colour comparisons.

Motion	Render	group1	group2	n1	n2	*p*	p.adj
R	PC1	C	M	40	40	1.00E+03	1.00E−03**
S	PC1	C	M	40	40	2.34E−13	2.34E−13****
R	PC8	C	M	40	40	4.00E−03	4.00E−03**
S	PC8	C	M	40	40	9.00E−03	9.00E−03**
R	PC35	C	M	40	40	1.60E−05	1.60E−05****
S	PC35	C	M	40	40	7.60E−02	7.60E−02
R	VX2	C	M	40	40	3.50E−02	3.50E−02*
S	VX2	C	M	40	40	1.07E−04	1.07E−04***
R	VX4	C	M	40	40	8.90E−01	8.90E−01
S	VX4	C	M	40	40	6.85E−04	6.85E−04***
R	VX6	C	M	40	40	4.35E−01	4.35E−01
S	VX6	C	M	40	40	3.00E−03	3.00E−03**

Significance indicated by *s for Bonferroni adjusted *p* values.

Simple simple pairwise comparisons were run for each motion and colour condition across all levels of the other conditions, *p* values were adjusted using Bonferroni correction. The results are shown in Table 5. Summaries of key statistics pertaining to the proposed hypotheses follow.

To address H4 and H5 we compare the monochrome-static condition with the colour-static and monochrome-rotation conditions for each render style at the different resolutions. For point clouds rotation has a highly significant effect on performance at all resolutions where as for voxels the effect significance decreases as resolution decreases. The significance of colour decreases with resolution for point clouds, with no significance at low resolution, but significance remains almost constant for voxels at all resolutions.

To address H7 we compare the rotation conditions across resolutions within each rendering style. There is no significant difference between point clouds in the rotation conditions at high and medium resolutions. There is a significant increase in the colour-rotation condition between the highest and lowest resolution. For voxels high resolution voxels perform significantly better than medium and low resolution voxels in both rotation conditions.

## 6 Discussion

The key finding from our initial analysis shows that there is a three-way interaction between our independent variables. Our followup analysis shows that the effect of both colour and motion is affected by how the objects are rendered, indeed render impacts performance for all combinations of colour and motion. This leads us to suppose that the utility of colour and motion is dependent on how the data is rendered. In order to investigate this supposition, and thus investigate our hypotheses, we conducted pairwise analysis for all conditions, across all levels of the other conditions.

It is clear from our findings that in the majority of cases objects are easier to recognise when rendered as point clouds than when rendered as voxels, particularly lower resolution voxels, providing support for H1 (*Point clouds will be easier to recognise than voxels.*). However, it is worth noting that in most cases high resolution voxels perform as well as higher resolution point clouds, and out perform low resolution point clouds in the majority of conditions. This suggests that at high resolution the utility of voxels might be leveraged in user interface design without sacrificing object recognition. At lower resolutions of voxels, object recognition is clearly a significant challenge, limiting their utility to cases where precise object identification is not necessary. They may have a use to reduce computational overhead for initial rendering of data, and areas requiring more detailed inspection could be tagged for re-rendering in a higher resolution format. Additionally, further work is required to investigate whether user training might make object identification easier at lower voxel resolutions.

Our findings only support H2 (*As the resolution of point clouds increases object recognition will improve.*) in the colour-static condition. There is only partial support for H2 in the monochrome-rotation condition as high resolution is no better than medium resolution, but both are better than low resolution. It seems plausible that there might be a ceiling effect on the benefit of increased point cloud resolution in some cases. In the monochrome-static condition there is no difference in performance across the resolutions. Looking at the pattern of results for the monochrome-static condition appears to have a floor effect, performing similarly in all render conditions. Counter intuitively in the colour-rotation condition performance improves as resolution decreases, further study is needed to find out why this is. Taken together the findings regarding H2 imply that provided motion cues are available high resolution is not vital.

Our findings provide only partial support for H3 (*As the resolution of voxels increases object recognition will improve.*) as high resolution voxels outperform medium and low resolution voxels in all conditions, but there is very little difference between medium and low resolution voxels. This reinforces our conclusion that lower resolution voxels are of limited utility.

The utility of colour decreases with resolution for point clouds, with no effect at low resolution, but remains almost static for voxels at all resolutions, largely refuting H4 (*Colour will have a greater impact for point clouds than for voxels.*) with the exception of high resolution point clouds where the effect is the strongest. For point clouds rotation has a large effect on performance at all resolutions where as for voxels the effect decreases as resolution decreases, supporting H5 (*Motion will have a greater impact for point clouds than for voxels.*). This implies that for point clouds, motion is the most important cue for object recognition, where as for voxels, motion or colour is sufficient for an improvement in object recognition.

For H6 (*Motion will compensate for a lack of colour.*) to be supported monochrome-rotation conditions must outperform monochrome-static conditions, and be close in performance to colour-static conditions. Our findings show that H6 is supported for voxels of all resolutions. However, our findings show that for point clouds this only holds true at high resolution, as monochrome-motion outperforms colour-static at medium and low resolutions, this provides partial support for H6, as the effect of motion exceeds the effect of colour for medium and low resolution point clouds. This implies that in the case of robot sensor data where colour information could not be collected, motion can be used to maintain scene understanding for users.

Our findings provide strong support for H7 (*Motion will compensate for reduced resolution in point clouds but not voxels.*) as motion is unable to improve the performance of voxels at low and medium resolutions, but is able to improve performance for medium and low resolution point clouds. By combining this with our finding that colour and motion effects are similar for voxels, this suggests that if lower resolution voxels need to be used other compensatory approaches are required, operator training is one possible approach that we aim to test in the future.

## 7 Limitations and further work

There are a number of limitations to our study that restrict the generalisability of our results to real digital twin virtual environments. Firstly the motion we have chosen is a pre-rendered exocentric rotation of the scene, and there are a large number of ways a particular user might navigate the VE to facilitate scene understanding. For example linear motion might be sufficient to elicit the kinetic depth effect, or motion to change the distance between the user and objects may have utility. Investigating the impact of different user motions, and perhaps a user study that aims to capture typical user behaviour are both promising avenues for future work. Such studies would aid in the design of tools to allow dynamic digital twin manipulation similar to those found in Lubos et al. (2014); Garrido et al. (2021), another area we plan to work on in the future.

Secondly, in order to have controlled conditions we have used artificially constructed scenes composed of individually scanned objects. Real world environments, particularly in our chosen application area of nuclear decommissioning, are likely to be far more cluttered. Further, by composing the VE from sensor data from multiple real robots the VE is likely to have a much higher incidence of noise. Performing a similar study using a large VE composed from real world data would allow testing of the applicability of our findings.

Finally, participants in our study received no prior training. This would not be the case were a teleoperation system built using a digital twin VE user interface be deployed in the real world. It would be instructive to see whether training and/or practice could improve object recognition for lower resolution data, particularly for voxels: as outlined in the introduction, high density colour point cloud data is not always available.

## 8 Conclusion

In this paper we have presented a user study to evaluate factors affecting recognition of digital twins of real world objects created from point cloud data. We have investigated render style, resolution, colour, and motion. These are factors that effect design decisions for robot sensing, data transmission, data rendering and User Interface (UI) design. This work contributes to the development of a teleoperation system for survey and maintenance robots in an unknown and/or dynamic environment, which will utilise onboard robot sensing to capture environmental data, such that a digital twin virtual environment can be constructed.

Our main findings were that objects in point cloud digital twins were easier to identify than voxels, and such objects could still be recognised at low resolutions provided motion cues were available; colour aided recognition for point clouds only when motion was also present. This has implications for the design of robot sensor suites and data gathering and transmission protocols. We posit that a useful PCVE could be rendered using data from lower resolution sensors such as Lidar which require lower communication bandwidth, and lower computational overhead to render. By moving around in such a PCVE an operator could direct the robots to gather additional sensor data on areas of interest, which could be incrementally rendered with higher densities of points.

Another important finding was that high resolution voxels had similar object recognition performance to higher resolution point clouds. This has important implications for use of voxels as a rendering style in digital twin teleoperation environments, as voxels can be more easily manipulated and tagged with information for mission planning (for example decommissioning procedures). Further, voxels have other benefits in delineating components of objects by lighting effects, and maintaining consistency regardless of viewer distance.

Finally motion and, to lesser extent, colour cues aided object recognition. Consequently robots should be endowed with colour capturing capability where possible, and more importantly, tools and training should be provided on digital twin VE teleoperationinterfaces to facilitate motion around objects that need to be identified and inspected.

Our results have application in any area where human data perception in a digital twin virtual environment (DTVE) is required, informing design decisions on rendering and UI design approaches. Further, our testing methodology can be adapted to facilitate analysis with different environments, robot sensing capabilities, rendering approaches *etc.* Further application of our testing methodology would improve understanding of the generalisability of our results and testing procedure.

## Data Availability

The datasets generated and analysed for this study can be found in the UWE Bristol data repository: PB. Robots for Nuclear Environments - Impact of Resolution, Colour, and Motion on Object Identification in Digital Twins from Robot Sensor Data. UWE, http://researchdata.uwe.ac.uk/673.

## Footnotes

1 We use these definitions to give us a common terminology to use between point clouds and voxel grids rather than suggesting that resolution is the most appropriate term to refer to these variations in object rendering.

2
www.Qualtrics.com

3
www.Prolific.co

4
http://wiki.ros.org/rtabmap_ros

5
https://assetstore.unity.com/packages/tools/utilities/point-cloud-free-viewer-19811

6 To aid legibility numerical results of the statistical tests have been left in the results tables and are not included in the text detailing the findings.
